# Clue Cells on Vaginal Wet Preparation Are Not Associated with Urinary Tract Infections or Positive Urine Cultures

**DOI:** 10.5811/westjem.2022.2.55000

**Published:** 2022-06-18

**Authors:** Johnathan Michael Sheele, Carolyn Mead-Harvey, Nicole Hodgson

**Affiliations:** * Mayo Clinic, Department of Emergency Medicine, Jacksonville, Florida; † Mayo Clinic, Division of Clinical Trials and Biostatistics, Phoenix, Arizona; ‡ Mayo Clinic, Department of Emergency Medicine, Phoenix, Arizona

## Abstract

**Introduction:**

Clue cells result from aberrant vaginal microflora and are associated with an increased vaginal pH, which can allow colonization of uropathogens in the vaginal introitus, increasing the risk for urinary tract infections (UTI). We sought to determine whether clue cells on vaginal wet preparation in the emergency department (ED) are associated with emergency physician diagnoses of UTIs and positive urine cultures.

**Methods:**

We conducted a retrospective analysis examining a dataset of women (≥18 years of age) who received both a genital wet preparation and urine testing in the ED. Both chi-square and multivariable regression analysis were performed.

**Results:**

We analyzed 14,952 encounters. On both univariable and multivariable analyses, emergency physicians diagnosed significantly fewer clue cell-positive women with a UTI (10.9% diagnosed with UTI vs 13.1% without UTI) (P <.001). Women with clue cells on vaginal wet preparation were not more likely to have a positive urine culture or have a urine culture growing *Escherichia coli*. Pregnant women with clue cells on vaginal wet preparation were not more likely to have a UTI or have a positive urine culture.

**Conclusion:**

Emergency physicians diagnosed significantly fewer women with UTIs when they found clue cells on vaginal wet preparation. Clue cells on vaginal wet preparation were not associated with an increased likelihood of a positive urine culture or having *E. coli* growing in the urine.

## INTRODUCTION

Genitourinary tract infections, especially urinary tract infections (UTI), are common in the emergency department (ED).^1^ Emergency physicians frequently perform vaginal wet preparation on women with genitourinary complaints, especially when there are concerns of vaginitis or sexually transmitted infections (STI). Vaginal wet preparation results can help risk-stratify ED patients for STIs.^2^

Bacterial vaginosis (BV) is the most common cause of vaginitis in outpatient medicine and results when anaerobic bacteria replace the normal lactobacilli colonizers of the vagina.^3^ Clue cells are vaginal epithelial cells covered in bacteria from an overgrowth of *Gardnerella vaginalis*, *Mycoplasma hominis*, *Mobiluncus* species, and *Peptostreptococcus* species.^3^ Clue cells on wet preparation represent a disruption of the vaginal microbiome and have about a 53–90% sensitivity and 40–100% specificity for BV.^4^ Some of the risk factors for BV overlap with UTI, including frequent sexual activity, use of spermicide, alterations of vaginal flora, and vaginal douching.^3^,^5^ Previous studies suggest that BV is associated with both UTI and positive urine culture.^6^–^11^ Some propose that a higher vaginal pH and fewer lactobacilli found with BV allow uropathogens to thrive in the vaginal introitus leading to increased risk for UTI and bacteriuria.^12^,^13^ Women with recurrent UTIs are more likely to have vaginal coliform bacteria, and this colonization often precedes bacteriuria.^14^

We sought to determine whether the presence of vaginal clue cells was associated with a diagnosis of UTI in the ED, and secondarily whether vaginal clue cells were associated with having a positive urine culture, having a urine culture grow *Escherichia coli*, and being diagnosed with a UTI while having a positive culture. We also examined these associations in pregnant women.^6^,^7^,^10^

## METHODS

We performed a secondary analysis of an existing dataset of ED encounters (N = 75,000) where patients ≥18 years of age received testing for gonorrhea, chlamydia or trichomonas, or received a urinalysis and urine culture. All ED visits occurred at University Hospitals between April 18, 2014–March 7, 2017. The dataset was created by University Hospitals information technology by extracting data from the institution’s electronic health records. For our study, we excluded men, women without a genital clue-cell result, and women without a urinalysis or a urine culture (Supplement 1). Data has previously been published from this dataset.^2^,^15^–^21^ The Mayo Clinic institutional review board provided an exemption from full review.

We categorized patients as being diagnosed with a UTI if they had a specific ED *International Classification of Diseases* (ICD) code (Supplement 1). We classified women as pregnant if they had a pregnancy-related ICD code or had a positive pregnancy test in the ED. For the urinalysis, we report the mean number of red blood cells (RBC) and white blood cells (WBC) if a range was provided, and all cells/high powered field (HPF) ≥101 were recoded as 101 cells/HPF. For the urine culture, we categorized urine cultures as positive (≥10,000 colony-forming units per milliliter [CFU/mL]), negative (0–10,000 CFU/mL), or not performed. Neither Amsel’s nor Nugent’s criteria for BV could be determined because no vaginal “whiff test,” Gram stain, or pH were recorded.

## STATISTICAL ANALYSIS

Categorical variables are presented as counts and percentages with chi square used to test associations. Continuous variables are presented as median and interquartile range with two-sample t-tests used to test associations. Multivariable logistic regression analysis was performed using clue cells as the dependent variable. We calculated odds ratios (OR) and 95% confidence intervals from the multivariable model. A *P*-value <.05 was considered statistically significant. Statistical analyses were conducted with statistical software JMP Pro 14 (JMP Statistic Discovery LLC, London, Ont, Canada) and SAS version 9.4 (SAS Institute, Inc. Cary, NC).

## RESULTS

There were 14,952 encounters included in the analysis (Supplement 1). We summarize patient characteristics and laboratory findings in Table 1. After adjusting for demographics and urinalysis, women with positive clue cells on vaginal wet preparation were significantly less likely to be diagnosed with a UTI (n = 707 [10.9%] vs n = 1115 [13.1%]; OR .75 [.66–.85]; *P* <.001) (Tables 1 and 2).

On univariable analysis, the secondary outcomes of women with clue cells on vaginal wet preparation, compared to those without clue cells:

1. if diagnosed with a UTI, were not more likely to have a positive urine culture (n = 123; 36.0%) vs (n = 235; 37.7%), respectively (OR .93 [.71–1.22]; *P* =.59).
2. if pregnant, were not more likely to be diagnosed with a UTI (n = 93; 6.6%) vs (n = 143; 7.6%), respectively (OR .87 [.66–1.13]; *P* =.29).
3. if pregnant and diagnosed with a UTI, were not more likely have a positive urine culture (n = 9; 17.6%) vs (n = 24; 27.0%), respectively (OR .58 [.25–1.37]; *P* =.21).
4. if pregnant, were not more likely to have a positive urine culture (n = 60; 14.3%) vs (n = 94; 16.2%), respectively (OR .87 [.61–1.23]; *P* =.43).

After adjusting for demographics and urinalysis we found that patients with vaginal clue cells were significantly less likely to have a urine culture performed (OR .85 [.78–.93]; *P* <.001) (Table 2). A clinical diagnosis of UTI was not a significant effect modifier of the association between vaginal clue cells and a positive urine culture (*P* = .72) (Supplement 2). Vaginal clue cells were not associated with having a urine culture of ≥10,000 CFU/mL bacteria (vs <10,000 CFU/mL) (OR 0.95 [0.79–1.16]; *P* = 0.64), nor with having a urine culture growing *E coli* (vs non-*E. coli*) (OR 1.15 (.73–1.61); *P* =. 41) (Supplement 3). Pregnancy was not a significant effect modifier of the association between wet prep clue cells and UTI (*P* = .67) or a positive urine culture (*P* = .10) (Supplement 2).

## DISCUSSION

Our data does not support the hypothesis that clue cells, which reflect a dysregulated vaginal microbiome, are associated with an increased risk for UTI or positive urine culture in the ED. Other studies finding BV to be associated with UTI included using bacteriuria as sole surrogate marker for UTI,^11^ focusing on pregnant women,^6^,^10^ examining women from gynecology clinics,^6^–^8^,^11^ or outside the United States^6^,^9^–^11^ where practice patterns may differ, or only used univariable statistics.^6^,^7^,^9^,^11^ Our findings appear unique in the medical literature, which could be related to the ED practice environment or to the more advanced analyses we present.

Our analysis does present a possible alternate cause for this finding. Not all women diagnosed with a UTI received a genital wet preparation or a urine culture, and not all women with a genital wet preparation received a urinalysis or urine culture, demonstrating selection bias (Supplement 1). Emergency physicians may have limited further testing after discovering clue cells on wet prep, prematurely anchoring and disregarding the possibility of concurrent BV and UTI. Clinicians were significantly less likely to order a urine culture when vaginal clue cells were identified, which supports this as a possible alternate explanation for our findings.

## LIMITATIONS

Our analysis did not include vaginal pH or vaginal uropathogenic culture results, and we could only use the presence of clue cells to identify the presence of a dysregulated vaginal microbiome.^22^ Neither Amsel’s nor Nugent’s criteria were available for analysis, and we did not compare our outcomes of interest to women diagnosed clinically with BV. Because we did not have the patients’ past medical histories of UTIs or any longitudinal data, we could not determine whether women with vaginal clue cells were more likely to get recurrent or future UTIs. Urinary tract infections and asymptomatic bacteriuria are frequently misdiagnosed and incorrectly treated in the ED, which could have affected the validity of the study findings, especially since our definition of a UTI was the ED diagnosis. One study found that BV was only associated with *E. coli* UTI in women who used a diaphragm, but our dataset did not include methods of contraception.^14^

## CONCLUSION

Previous studies identified a dysregulated vaginal microbiome to be associated with an increased risk for UTI. However, we found that vaginal clue cells in the ED were associated with a significantly reduced risk of being diagnosed with a UTI. Vaginal clue cells in the ED were not associated with an increased likelihood of having a positive urine culture, having a urine culture growing *E coli*, being diagnosed with a UTI and having a positive urine culture, being pregnant and having a UTI, or being pregnant with a positive urine culture.

## Supplementary Information







## Figures and Tables

**Table 1:** Encounter characteristics.

	Total (N = 14,952)
Age (years), median (IQR)	26 (22.2, 32.4)
Black/African American, n (%)	13,191/14,890 (88.6%)
Marital status married/life partner, n (%)	1,389/14,910 (9.3%)
Pregnant, n (%)	3,298 (22.1%)
Discharged from ED, n (%)	14,062 (94.0%)
*Neisseria gonorrhea* NAAT positive, n (%)	436/14,556 (3.0%)
*Chlamydia trachomatis* NAAT positive, n (%)	1,146/14,544 (7.9%)
*Trichomonas vaginalis* NAAT positive, n (%)	371/4,428 (8.4%)
Red blood cells, n; median (IQR)	10,712; 2.5 (2.0, 12.5)
White blood cells, n; median (IQR)	10,714; 5.0 (2.5, 13.0)
Wet prep-clue cells present, n (%)	6,469 (43.3%)
Wet prep-yeast cells present, n (%)	949/14,739 (6.4%)
Bacteria present, n (%)	6506/10,730 (60.6%)
Blood present, n (%)	5848/14,770 (39.6%)
Leukocyte esterase present, n (%)	6753/14,760 (45.8%)
Mucous present, n (%)	6314/10,722 (58.9%)
Nitrite positive, n (%)	558/14,861 (3.8%)
pH, n; median (IQR)	14,865, 6.0 (5.0, 6.0)
Protein present, n (%)	4341/14,850 (29.2%)
Urobilinogen (2.0+), n (%)	3604/14,865 (24.2%)
WBC clumps present, n (%)	476/10,623 (4.5%)
Yeast in urine present, n (%)	284/10,667 (2.7%)
Urine culture CFU/mL ≥10,000, n (%)	893/4,505 (19.8%)
Diagnosed with a urinary tract infection (UTI) in the ED, n (%)	1,822 (12.2%)
Urine culture positive, n (%)	358/965 (37.1%)
*E. coli*-positive urine culture, n (%)	562/878 (64.0%)

*IQR*, interquartile range; *ED*, emergency department; *NAAT*, nucleic acid amplification test; *WBC*, white blood cell; *CFU*, colony-forming units; *mL*, milliliter; *E. coli*, Escherichia coli.

**Table 2:** Multivariable regression model examining the association of vaginal clue cells on vaginal wet preparation.

Variable	OR (95% CI)	P-value
Age (years)	0.99 (0.99, 1.00)	<0.001
Black/African American (vs other)	2.17 (1.89, 2.50)	<0.001
Married/life partner (vs. other marital status)	0.77 (0.66, 0.90)	<0.001
Pregnant (vs not pregnant)	0.93 (0.84, 1.03)	0.16
Urine:		
Bacteria (0–4+)	1.17 (1.13, 1.22)	<0.001
Blood (0–3+)	1.05 (1.00, 1.09)	0.04
Leukocyte esterase (0–3+)	1.02 (0.97, 1.06)	0.52
Mucus (0–4+)	0.94 (0.92, 0.97)	<0.001
pH (5–9)	1.03 (0.99, 1.08)	0.17
RBCs (0–101 cells/HPF)	0.99 (0.99, 0.99)	<0.001
WBCs (0–101 cells/HPF)	1.00 (1.00, 1.00)	0.65
WBC clumps present (vs. absent)	1.09 (0.88, 1.35)	0.45
Nitrite positive (vs. absent)	1.11 (0.92, 1.34)	0.29
Protein positive (vs. negative)	1.31 (1.20, 1.44)	<0.001
Trichomonas present (vs. absent)	1.31 (1.01, 1.70)	0.04
Diagnosed with a UTI (vs. no UTI diagnosis)	0.75 (0.66, 0.85)	<0.001
Urine culture ordered (vs. no urine culture done)	0.85 (0.78, 0.93)	<0.001

*OR*, odds ratio; *CI*, confidence interval; *RBCs*, red blood cells; *HPF*, high powered field; *WBCs*, white blood cells; *UTI*, urinary tract infection.
