# Supplementary material for: Clue Cells on Vaginal Wet Preparation Are Not Associated with Urinary Tract Infections or Positive Urine Cultures
Source: West J Emerg Med. 2022 Jun 18;23(4):468–72. doi: 10.5811/westjem.2022.2.55000 (PMC9391019; doi:10.5811/westjem.2022.2.55000)
Supplement: Supplementary file 1 [file wjem-23-468-s001.docx]

Supplement 1. Description of patients included and excluded from the dataset.

Dataset (n=75,000)

19,308 Male

55,692 Female

38,596 Females with no documented vaginal clue cell result

16,736 Females with a documented vaginal clue cell result

1,784 No or insufficiently documented urinalysis or urine culture

14,952 Documented urinalysis or urine culture

Within the complete dataset (n=75,000) there were:

11,384 women diagnosed with a UTI

9,829 women diagnosed with a UTI and received a urine culture

956 women diagnosed with a UTI, received a urine culture, and had a vaginal wet preparation clue cell result

16,736 women had a vaginal wet preparation clue cell result

4,505 women had a vaginal wet preparation clue cell result and had a urine culture performed

1,839 women had a vaginal wet preparation clue cell result and were diagnosed with a UTI

38,164 women had a urine culture performed

27,516 women had urine cultures growing 0-10,000 CFU/mL

10,648 women had urine cultures growing ≥10,000 CFU/mL

4,974 women were pregnant

429 pregnant women diagnosed with a UTI

296 pregnant women diagnosed with a UTI and had a urine culture performed

1,935 pregnant women had a urine culture performed

3,605 pregnant women had a vaginal wet preparation clue cell result

238 pregnant women who had a vaginal wet preparation clue cell result and were diagnosed with a UTI

For our analysis we only included women that had a vaginal wet preparation clue cell result and who either had a urinalysis or urine culture performed (n=14,952):

1,822 women diagnosed with a UTI

965 women diagnosed with a UTI and received a urine culture

4,505 women had a urine culture performed

3,612 women had urine cultures growing 0-10,000 CFU/mL

893 women had urine cultures growing ≥10,000 CFU/mL

3,298 Pregnant women

236 Pregnant women diagnosed with a UTI

140 Pregnant women diagnosed with a UTI and had a urine culture performed

1,001 Pregnant women who had a urine culture performed

Patients were considered to have an ED diagnosis of UTI if they had one of the *International Classification of Diseases, Ninth Revision, Clinical Modification* (ICD-9-CM) or *Tenth Revision, Clinical Modification* (ICD-10-CM) codes at ED discharge: N30.90, O86.22, N30.00, N30.91, N30, N30.0, N30.01, N30.9, O23.10, O86.20, N39.0, O08.83, O03.38, O04.88, O03.88, O86.2, O86.29, O23.40, 646.64, 599.0, 639.8, 646.60, 595.0, 595.9, 595.89, or 595.0.

Women were pregnant if they had or had a positive pregnancy test in the ED or an ICD code: O00.1, O00.8, O21.9, O00.90, Z32.01, O00, O20, 643.93, 633.90, V72.42.
